# Honey Mitigates Radiation-Induced Oral Mucositis in Head and Neck Cancer Patients without Affecting the Tumor Response

**DOI:** 10.3390/foods6090077

**Published:** 2017-09-06

**Authors:** Suresh Rao, Sanath K. Hegde, Pratima Rao, Chetana Dinkar, Karadka R. Thilakchand, Thomas George, Manjeshwar P. Baliga-Rao, Princy L. Palatty, Manjeshwar S. Baliga

**Affiliations:** 1 Department of Radiation Oncology, Mangalore Institute of Oncology, Mangalore 575002, India; raos_64@yahoo.com (S.R.); Sanathkumarhegdemio@yahoo.com (S.K.H.); 2 Department of Dentistry, Mangalore Institute of Oncology, Mangalore 575002, India; rathimarao@gmail.com (P.R.); chetanadinkar.MIO@yahoo.com (C.D.); 3 Department of Anesthesiology, Karnataka Institute of Medical Sciences, Hubballi 580022, India; thilak91@gmail.com; 4 Summer Research Fellow, Mangalore Institute of Oncology, Mangalore 575002, India; jeffthomasgeorge@gmail.com; 5 Department of Hospital Pharmacy, Mangalore Institute of Oncology, Mangalore 575002, India; poonam.baliga.rao@gmail.com; 6 Department of Pharmacology, Father Muller Medical College, Mangalore 575002, India; drprincylouispalatty@gmail.com

**Keywords:** honey, radiation, mucositis, treatment response, cell kill

## Abstract

Radiation-induced mucositis is a dose-limiting factor in the effective treatment of head and neck (H & N) cancers. The objective of this study was to understand the efficacy of honey in mitigating radiation-induced mucositis and whether it would interfere with tumor control. This was a single-blinded, randomized, controlled study and was carried out in patients with H & N cancer requiring curative radiotherapy (>62 Gy (Gray)). The patients meeting the inclusion criteria were randomly assigned to receive either honey (*n* = 25) or povidone-iodine (active comparator) (*n* = 25) during radiotherapy. Oral mucositis was assessed using the RTOG (Radiation Therapy Oncology Group) grading system before the start, during, and at the end of the treatment by an investigator unaware of the treatment. The results indicate that when compared with the active comparator, honey reduced the radiation-induced oral mucositis, decreased the incidence of intolerable mucositis, treatment breaks, loss of treatment days (*p* < 0.0001 and < 0.0003) and did not affect the radiation-induced tumor response. The clinical observations indicate that honey mitigates the radiation-induced mucositis and does not interfere with tumor cell killing.

## 1. Introduction

Mucositis is an unavoidable side effect observed in most cancer patients undergoing radiotherapy for head and neck cancers (H & N) [1]. Depending on the severity, oral mucositis is classified as tolerable (grade 1 and 2 mucositis) and intolerable mucositis (grade 3 or 4) [1]. In severe conditions, mucositis also contributes to local and systemic infections [1]. This will affect the treatment schedule, dose, and therapeutic outcome [1]. From a therapeutic perspective, there are no drugs for the avoidance/mitigation of radiation-induced mucositis and preventive procedures consist of symptom management and adherence to basic oral care to prevent infections and alleviate the mucosal symptoms [1]. In most hospitals colloidal silver solutions, salt and soda rinses, or hydrogen peroxide rinses are used [1]. In recent years, the recombinant human keratinocyte growth factor-1 (KGF-1) (palifermin) has also been reported to be useful in mitigating mucositis [1]. However, its exorbitant cost is a major deterrent from widespread use in developing countries [1].

In the recent past, studies from around the world have shown that honey was effective at mitigating radiation- and chemotherapy-induced mucositis [2,3,4,5,6,7,8,9,10,11,12,13,14,15]. Met analyses have substantiated the observed beneficial effects [13,14,15]. Honey is arguably one of the world’s oldest dietary agents with medicinal use, and scientific studies have shown it to possess wound healing, antimicrobial, analgesic, and anti-inflammatory effects in various experimental studies [2,3,4,5,6,7,8,9,10,11,12,13,14,15]. The present study was principally carried out to observe whether honey interfered with the radiation-induced tumor response while having a positive effect against oral mucositis.

## 2. Patients and Methods

### 2.1. Patient Population

The study was carried out with histopathologically confirmed H & N cancer patients requiring radiotherapy. Patients who had radical surgery prior to six weeks at the start of radiation treatment were also included. The exclusion criteria included patients younger than 18 years of age; women who were pregnant or lactating; patients on high doses of non-steroidal anti-inflammatory drugs; poor oral hygiene and xerostomia; and significant comorbidity such as poorly controlled diabetes mellitus, hypertension, schizophrenia, bipolar disorder, and severe depression. Patients who had had oral surgery less than six weeks previously, those who had had chemotherapy within the past six weeks, and those who had previously been treated with radiotherapy for H & N cancers were excluded from the study. The study was approved by the Hospital Ethics Committee (MIO/IEC/2010/12/05). Before the start of the study, patients and their caregivers were informed about the treatment schedule, the benefits, and the possible adverse effects of the study by a trained professional. They were also informed of their right to withdraw during the course of the study. The queries from both patients and caregivers were answered and written informed consent was obtained from the patients.

### 2.2. Study Design

This was a randomized, investigator-blinded clinical study conducted at Mangalore Institute of Oncology from January 2012 to June 2012. A sample size of 50 subjects was considered from previous studies [7,11]. Before initiation of the study, the volunteers were examined for decayed teeth, ulcers, or lesions in the oral mucosa by an orodental physician. Of the eligible 56 patients, six did not meet the inclusion criteria on account of metabolic disease (four) or very poor oral hygiene (two). The remaining 50 volunteered to participate in the study. The randomization was performed using opaque envelopes by a staff member unaware of whether they were distributing the active comparator (povidone-iodine Group A) or the test group (honey Group B).

### 2.3. Radiation Treatment

The curative radiation treatment dose (62–70 Gy (Gray)) was planned as per the international guidelines [16] using the linear accelerator (Varian Medical Systems, Palo Alto, CA, USA). All patients received a fraction of 2 Gy, five consecutive days a week, for seven consecutive weeks). Some patients also received carboplatin infusion (70 mg/m^2^/day intravenously (IV)) prior to the scheduled radiation. The patients were provided with the standard oral, dental, medical, and supportive care [16]. A feeding tube was placed only when necessary. Routine screening was performed every other day for oral infections and antimicrobial agents were prescribed based on the culture sensitivity reports. The participants were informed to clean their teeth thrice a day (early morning, after lunch, and at night) using a soft toothbrush. Patients with spontaneous gum bleeds were provided with cleaning solutions. As all patients were in the hospital during the treatment period it was easy to monitor their adherence to diet, medications, practice of oral hygiene, and the interventional agents.

### 2.4. Study Mouthwashes

In this study, group A were assigned to povidone-iodine (Betadine 1 mL and 100 mL water), while those in group B were allocated to honey (Dabur India, New Delhi, India). The honey used in the study was of polyfloral origin. To avoid between-batch variation, both honey and povidone-iodine were purchased in a single lot and used for the study. The patients in the povidone-iodine group were asked to follow the schedule as per the protocol of Madan and co-workers [17]. The patients in the honey cohort were taught to apply honey three times a day (1 h prior to radiation, and 2 and 6 h after radiation). The patients were taught to swish the oral cavity and to eat at least 30 minutes after the honey treatment.

### 2.5. Outcome Measures

The assessment for the degree of mucositis was done first at the time of mold preparation and then on a weekly basis during the course of radiation treatment. The objective scoring was done by a senior orodental pathologist unaware of the intervention received by each patient. A regular torchlight was used to grade the degree of mucositis in the oral cavity, while a laryngoscope was used to assess the oropharyngeal areas. The grading was scaled from 0 to 4, depending on the severity of oral mucositis based on the The radiation therapy oncology group (RTOG) guidelines. Grades 0, 1, and 2 were “tolerable” and grades 3 and 4 were “intolerable” forms of mucositis, as described earlier [5]. The calibration of assessment was not required because the blinded researcher evaluated the patients throughout the study period [5].

### 2.6. Treatment Response Evaluation after Treatment Completion

The response to radiotherapy was assessed during the first follow-up (i.e., four weeks after completion of treatment). The clinical assessment was done by a senior radiation oncologist in accordance to the guidelines prescribed by World Health Organization [18]. Degree of tumor volume shrinkage was considered an index of radio responsiveness. Patients with 100% regression of tumor at the primary site were considered complete responders (CR), whereas partial responders (PR) had a higher than 50% regression and non-responders (NR) had a lower than 50% regression [18].

### 2.7. Statistical Analysis

Analysis of variance (ANOVA) was used to compare the extent of severe mucositis score on a weekly basis, testing the equality of proportion for the delay in incidence and the number of tolerable and intolerable mucositis, while the *χ*^2^ test was used to compare the total incidence of worst-ever grades of ulceration, the number of treatment days lost due to intolerable mucositis, weight loss, and tumor response. A *p* value < 0.05 was considered significant.

## 3. Results

### Clinical Study

**Figure 1:**
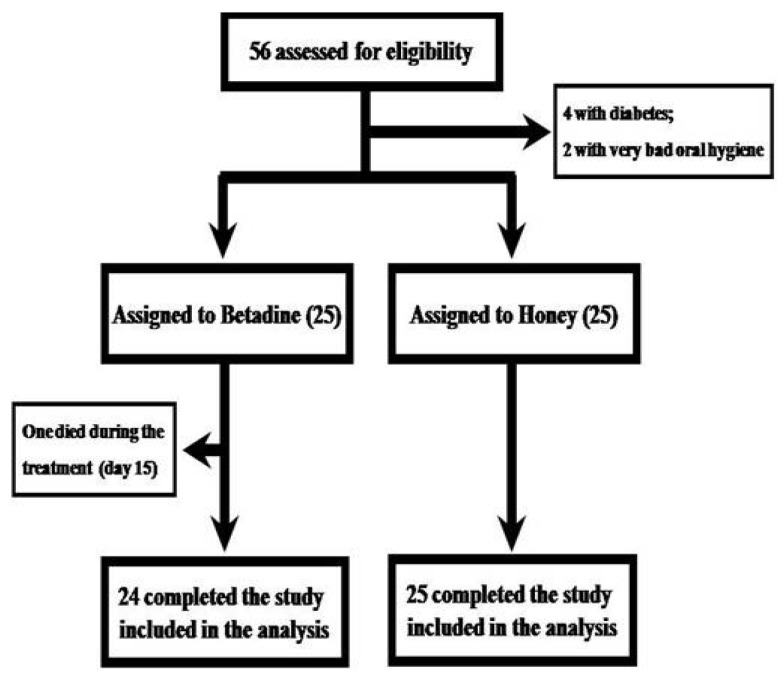
Patient flow in the randomized controlled study.

The details on age, sex, location of cancer, stage of cancer, type of treatment used, dose, incidence of breaks, and loss of days due to mucositis for the two groups are listed in Table 1 and Table 2. The study population consisted of 13 women and 37 men, with ages ranging from 34 to 73 years (56.29 ± 10.3). The mean age for women was 49.92 ± 9.34 (range 36–67), while that for men were 58.31 ± 10.69 (range 34–73). One patient in the control group succumbed to the disease during the third week of the treatment (Figure 1). The remaining 24 patients in the control group and 25 in the honey group continued with the planned treatment (Figure 1).

**Figure 2:**
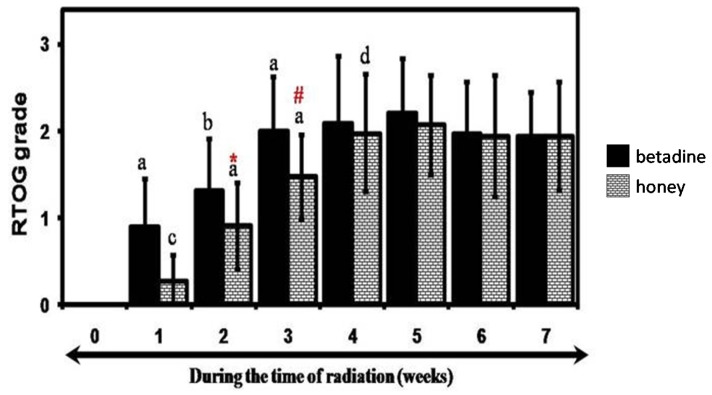
Severity of radiation-induced mucositis in the betadine (solid) and honey (bricked) cohorts during the course of the radiation treatment. Friedman’s test: a, *p* < 0.0001; b, *p* < 0.001; c, *p* < 0.014; d, *p* < 0.002; comparison with preceding week of the respective group and Mann–Whitney test. * *p* < 0.0001; # *p* < 0.003 (comparing between control and honey). RTOG, radiation therapy oncology group.

Radiation exposure caused mucositis in both the cohorts, and the mean mucositis scores are represented on a weekly basis in Figure 2. The onset of tolerable and intolerable mucositis was delayed in the patients using honey and was significant for week 2 (*p* < 0.0001) and 3 (*p* < 0.003). The early signs of radiation-mucositis were first seen at the end of first week after exposure to 10 Gy (5 fractions) in both the groups (Figure 2). At the end of the first week, 84% of the patients in the control and 24% in the honey group showed signs of grade 1 mucositis (*p* < 0.05). With continuation of the treatment, the incidence of grade 2 mucositis appeared only in the controls (32% (8/25) vs. 0% (0/25)) and was significant (*p* = 0.002) at week 2. The incidence of intolerable mucositis (only grade 3) were seen earlier and at the end of week 3 in controls (16% (4/24), *p* = 0.03) and reached a peak in both the groups at the end of week 7 (12/24 in controls vs. 8/25 in honey (*p* = 0.2)). There was no incidence of grade 4 mucositis in either group.

With respect to the number of treatment days lost, of the 25 patients in the honey group, three (15%) experienced treatment interruption between the 4th and 5th week of radiation. The corresponding number for patients in the povidone-iodine group was five (20.83%), of whom two needed a treatment break at an early stage before completion of 40 Gy (four weeks) and three after completing 40 Gy between the 5th and 7th weeks. The number of treatment days lost were 7.4 ± 0.08 and 6.33 ± 0.94 days, respectively, for the povidone-iodine and honey groups, and was not significant (Table 2). Additionally, it was observed that when compared to the povidone-iodine (4.86 ± 1.73) group, the weight loss was less in the honey cohorts (2.77 ± 0.85) and was statistically significant (*p* < 0.001).

With respect to assessing the effect of honey on radiation-induced cell killing and tumor regression, the clinical analysis performed four to five weeks after the last fraction of radiation showed that there was no significant difference in the observed cases of the complete response (CR), partial response (PR), and no response (NR). Cumulative results indicated that in the control group 45.8% (11/24) of CR, 25% (6/24) of PR, and 29.2% (7/24) NR were observed, while in the honey-treated group it was 52% (13/25) of CR, 20% (5/25) of PR, and 28% (7/25) of NR and was not significant.

## 4. Discussion

From a clinical perspective, the resulting inflammation and ulceration seen in mucositis are very painful [1]. The denuded epithelium can also provide access to the oral microbial flora and the resulting infection aggravates the condition [1]. Additionally, exposure to fractionated doses of radiation compromises the wound healing process and this aggravates and complicates the situation [1]. In the present study honey was observed to be effective at mitigating radiation mucositis, which is in agreement to earlier reports [2,3,4,5,6,7,8,9,10,11,12,13,14,15]. The observed effects could be attributed to the honey’s analgesic [9,12], antimicrobial [12], and wound-healing effects [19,20].

At a cellular level, exposure to ionizing radiation causes the generation of free radicals (reactive oxygen species and reactive nitrogen species), DNA strand breaks, and activation of transcription factors (NF-κB) [1]. Additionally, the immune cells also produce pro-inflammatory cytokines (Tumor necrosis factor-α (TNF-α), IL-1, and IL-6), which aggravate tissue injury and cell death [1]. Honey has been shown to possess free radical scavenging effects in various experimental systems of study; this could have contributed to the observed protective effects, at least in part [19,20].

The most important observation of our study was that, while honey was effective in mitigating radiation-induced mucositis, it did not interfere with the treatment response in H & N cancer patients.

## 5. Conclusions

The results of this study indicate that honey mitigates radiation-induced mucositis and that the protective effect does not interfere with tumor cell killing. The cell culture details indicate that treatment with honey enhanced the radiation-induced cell killing. Further studies are planned to validate these observations with appropriate study models, controls, and end point assays.

## Figures and Tables

**Table 1:** Patient details and tumor characteristics.

	Povidone-Iodine	Honey
Age (years)	55.8 ± 10.8	54.1 ± 11.3
Gender		
Male	17	20
Female	8	5
Site		
Alveolus	0	2
Buccal mucosa	4	3
Floor of the Mouth	0	2
Gingivobuccal sulcus	1	0
Palate	1	1
Pyriform sinus	0	2
Pharynx (Oro and Hypo)	0	1
Retromolar trigone	0	2
Tongue	17	12
Tonsil	2	0
Tumor/Node/Metastasis (TNM) stage		
Primary		
T1	2	8
T2	9	8
T3	8	5
T4	5	3
TX	1	1
Regional nodes		
N0	3	5
N1	13	11
N2	1	0
N2a	4	3
N2b	0	3
N2c	2	1
N3	1	2
NX	1	0
Metastasis		
M0	0	0
MX	0	0

**Table 2:** Treatment details and clinical end points.

Treatment Details	Povidone-Iodine	Honey
Radiation only	2	1
Chemoradiation	23	24
Dose of radiation	67.0 ± 2.3	68.1 ± 2.3
Death during the course of radiation treatment	1	0
Incidence of treatment breaks	5/24	3/25
Number of days	7.4 ± 0.8	6.33 ± 0.95
Appearance of mucositis	7.24 ± 3.10	10.16 ± 2.35 *
Treatment response details		
Complete response	45.8% (11/24)	52% (13/25)
Partial response	25% (6/24)	20% (5/25)
No response	29.2% (7/24)	28% (7/25)

* *p* = 0.027.

## References

[1] Sonis S.T. (2013). Oral mucositis in head and neck cancer: Risk, biology, and management. Am. Soc. Clin. Oncol. Educ. Book.

[2] Biswal B.M., Zakaria A., Ahmad N.M. (2003). Topical application of honey in the management of radiation mucositis: A preliminary study. Support. Care Cancer.

[3] Motallebnejad M., Akram S., Moghadamnia A., Moulana Z., Omidi S. (2008). The effect of topical application of pure honey on radiation-induced mucositis: A randomized clinical trial. J. Contemp. Dent. Pract..

[4] Rashad U.M., Al-Gezawy S.M., El-Gezawy E., Azzaz A.N. (2009). Honey as topical prophylaxis against radiochemotherapy-induced mucositis in head and neck cancer. J. Laryngol. Otol..

[5] Khanal B., Baliga M., Uppal N. (2010). Effect of topical honey on limitation of radiation-induced oral mucositis: An intervention study. Int. J. Oral Maxillofac. Surg..

[6] Maiti P.K., Ray A., Mitra T.N., Jana U., Bhattacharya J., Ganguly S. (2012). The effect of honey on mucositis induced by chemoradiation in head and neck cancer. J. Indian Med. Assoc..

[7] Jayachandran S., Balaji N. (2012). Evaluating the effectiveness of topical application of natural honey and benzydamine hydrochloride in the management of radiation mucositis. Indian J. Palliat. Care.

[8] Abdulrhman M., El Barbary N.S., Ahmed Amin D., SaeidEbrahim R. (2012). Honey and a mixture of honey, beeswax, and olive oil-propolis extract in treatment of chemotherapy-induced oral mucositis: A randomized controlled pilot study. Pediatr. Hematol. Oncol..

[9] Samdariya S., Lewis S., Kauser H., Ahmed I., Kumar D. (2015). A randomized controlled trial evaluating the role of honey in reducing pain due to radiation induced mucositis in head and neck cancer patients. Indian J. Palliat. Care.

[10] KobyaBulut H., GüdücüTüfekci F. (2016). Honey prevents oral mocositis in children undergoing chemotherapy: A quasi-experimental study with a control group. Complement. Ther. Med..

[11] Jayalekshmi J.L., Lakshmi R., Mukerji A. (2016). Honey on oral mucositis: A randomized controlled trial. Gulf J. Oncol..

[12] Al Jaouni S.K., Al Muhayawi M.S., Hussein A., Elfiki I., Al-Raddadi R., Al Muhayawi S.M., Almasaudi S., Kamal M.A., Harakeh S. (2017). Effects of honey on oral mucositis among pediatric cancer patients undergoing chemo/radiotherapy treatment at King Abdulaziz University Hospital in Jeddah, Kingdom of Saudi Arabia. Evid.-Based Complement. Altern. Med..

[13] Xu J.L., Xia R., Sun Z.H., Sun L., Min X., Liu C., Zhang H., Zhu Y.M. (2016). Effects of honey use on the management of radio/chemotherapy-induced mucositis: A meta-analysis of randomized controlled trials. Int. J. Oral Maxillofac. Surg..

[14] Co J.L., Mejia M.B., Que J.C., Dizon J.M. (2016). Effectiveness of honey on radiation-induced oral mucositis, time to mucositis, weight loss, and treatment interruptions among patients with head and neck malignancies: A meta-analysis and systematic review of literature. Head Neck.

[15] Cho H.K., Jeong Y.M., Lee H.S., Lee Y.J., Hwang S.H. (2015). Effects of honey on oral mucositis in patients with head and neck cancer: A meta-analysis. Laryngoscope.

[16] Elad S., Bowen J., Zadik Y., Lalla R.V. (2013). Development of the MASCC/ISOO Clinical Practice Guidelines for Mucositis: Considerations underlying the process. Support. Care Cancer.

[17] Madan P.D., Sequeira P.S., Shenoy K., Shetty J. (2008). The effect of three mouthwashes on radiation-induced oral mucositis in patients with head and neck malignancies: A randomized control trial. J. Cancer Res. Ther..

[18] World Health Organization (1979). WHO Handbook for Reporting Results of Cancer Treatment.

[19] Erejuwa O.O., Sulaiman S.A., Ab Wahab M.S. (2012). Honey: A novel antioxidant. Molecules.

[20] Samarghandian S., Farkhondeh T., Samini F. (2017). Honey and health: A review of recent clinical research. Pharmacogn. Res..

